# Mosquito-Borne Viruses and Suppressors of Invertebrate Antiviral RNA Silencing

**DOI:** 10.3390/v6114314

**Published:** 2014-11-11

**Authors:** Scott T. O’Neal, Glady Hazitha Samuel, Zach N. Adelman, Kevin M. Myles

**Affiliations:** Fralin Life Science Institute and Department of Entomology, Virginia Tech, Blacksburg, VA 24061, USA; E-Mails: onealst@vt.edu (S.T.O.); glady82@vt.edu (G.H.S.); zachadel@vt.edu (Z.N.A.)

**Keywords:** RNA silencing, RNAi, suppressor, mosquito, invertebrate, virus, arbovirus

## Abstract

The natural maintenance cycles of many mosquito-borne viruses require establishment of persistent non-lethal infections in the invertebrate host. While the mechanisms by which this occurs are not well understood, antiviral responses directed by small RNAs are important in modulating the pathogenesis of viral infections in disease vector mosquitoes. In yet another example of an evolutionary arms race between host and pathogen, some plant and insect viruses have evolved to encode suppressors of RNA silencing (VSRs). Whether or not mosquito-borne viral pathogens encode VSRs has been the subject of debate. While at first there would seem to be little evolutionary benefit to mosquito-borne viruses encoding proteins or sequences that strongly interfere with RNA silencing, we present here a model explaining how the expression of VSRs by these viruses in the vector might be compatible with the establishment of persistence. We also discuss the challenges associated with interrogating these viruses for the presence of suppressor proteins or sequences, as well as the candidates that have been identified in the genomes of mosquito-borne pathogens thus far.

## 1. Invertebrate Antiviral Immune Response

Infection of vertebrates with pathogenic organisms elicits two distinct immune responses. The first line of defense is an innate response, initiated by well conserved pathogen-associated molecular patterns (PAMPs). This is followed by an adaptive response that provides specificity and protection from future infections by similar pathogenic organisms. Although lacking the antibody-driven adaptive responses of vertebrate organisms, the invertebrate immune response to viral infection also demonstrates remarkable specificity, and possibly even memory of prior exposures, as suggested by one recent study [1]. This functional conservation is achieved through the action of small RNA pathways, which appear to comprise the primary antiviral defense of invertebrate organisms [2]. As a result of studies conducted primarily in the model organism *Drosophila melanogaster*, three classes of small RNAs have been identified in dipterans: small interfering RNAs (siRNAs), microRNAs (miRNAs), and piwi-interacting RNAs (piRNAs) [3].

## 2. siRNAs

The siRNA pathway appears to provide the primary antiviral immune response in invertebrate organisms [2]. The response is triggered by double-stranded RNA (dsRNA), which is produced by nearly all viruses as a byproduct of replication or gene expression [4]. The ribonuclease Dicer 2 (Dcr2) processes viral dsRNA into siRNA fragments 21 nucleotides (nt) in length. These siRNAs are then loaded into an RNA-induced silencing complex (RISC) with the help of the double-stranded RNA binding protein (dsRBP) R2D2 [5,6]. Once loaded into the RISC, one strand of the siRNA duplex is retained as a guide stand while the other, known as the passenger strand, is cleaved by the slicer activity of the enzyme Argonaute 2 (Ago2) and then removed by the endoribonuclease C3PO [7,8]. Finally, the 3’-terminal nucleotide of the remaining guide strand is 2’-O-methylated by the methyltransferase Hen1, fully activating the mature RISC [9]. Using the guide strand as a template, the RISC is able to slice complementary viral RNAs within the cell, effectively silencing viral gene expression. In addition to exogenous siRNA production, siRNAs can be produced from endogenously transcribed RNAs [10] and have been shown to target both transposable elements as well as transcripts from protein-coding genes [11]. Similarly, a study recently demonstrated that cDNAs are generated from viral RNAs during the early stages of infection, presumably by the reverse transcriptase activity of retrotransposons [1]. The authors of this study suggest that these cDNAs constitute a primitive form of immunological memory through the production of cDNA transcript-derived siRNAs that target the virus [1]. The authors speculate that these small RNAs may play a role in the establishment of persistence [1].

## 3. miRNAs

The second class of small RNAs is composed of miRNAs, which are processed from endogenously encoded hairpin transcripts known as primary miRNAs (pri-miRNAs). Drosha and the dsRBP Pasha process the pri-miRNA transcripts, yielding precursor miRNAs (pre-miRNAs) [12,13]. The pre-miRNAs are then bound and processed by Dicer 1 (Dcr1) and the dsRBP Loquacious, resulting in double-stranded miRNA duplexes of approximately 22 nt in length [14,15]. The duplex is then loaded into a RISC that contains Argonaute 1 (Ago1) [16]. This process differs from the loading of siRNAs into the RISC in several respects, most notably in that cleavage of the passenger strand is not necessary for maturation of the miRNA RISC [17], nor are the miRNAs 2’-O-methylated [9]. The miRNA pathway is primarily responsible for regulating gene expression, though several studies suggest that miRNAs may regulate the innate immune responses of invertebrates, consequently playing an indirect role in the response to viral infection [18]. Of note, a recent study offers intriguing evidence of a dengue virus (DENV; *Flaviviridae*: *Flavivirus*) type 2-encoded small RNA resembling a miRNA that may be involved in regulating its replication [19]. Analysis of other DENV serotypes has not produced evidence of conservation, and thus the relevance of these findings may be limited to DENV type 2 [20]. Nevertheless, this remains an interesting mechanism for potential modulation of the virus in the vector host.

## 4. piRNAs

The third class of small silencing RNAs consists of piRNAs, which range in size from 25 to 30 nt and associate with proteins in the Piwi clade of the Argonaute (Ago) family of proteins [21,22]. The biogenesis of piRNAs is thought to occur through one of two pathways, resulting in either primary or secondary piRNAs. Rather than the dsRNA substrates from which siRNAs and miRNAs are derived, the production of primary piRNAs is believed to occur from long, single-stranded RNAs (ssRNA) that are transcribed from discrete genomic loci termed piRNA clusters [21]. Although piRNA biogenesis pathways have not yet been fully elucidated, evidence suggests that these long ssRNA transcripts are processed by the endoribonuclease Zucchini to produce a pool of 5’ monophosphorylated precursor piRNAs (pre-piRNAs) [23,24]. The piRNA 3’ ends are likely formed by exonucleolytic trimming after the pre-piRNA has been loaded into the appropriate Piwi protein [25]. Similar to siRNAs, the final step appears to be 2’-O- methylation by Hen1, resulting in a mature piRNA-RISC complex [9].

Unlike siRNAs and miRNAs, Dicer does not appear to be involved in the generation of piRNAs [26,27]. Rather, small RNA profiling studies have demonstrated a 10 nt overlap at the 5’ ends of sense and antisense piRNAs, as evidenced by a bias for uridine at the 5’ end of antisense strands with a corresponding bias for adenine at the 10th position of sense strands [21,22]. Based on these observations, a “ping-pong” model has been proposed for the biogenesis of secondary piRNAs in which sense strands guide the production of antisense strands and vice versa, establishing an amplification loop [21,22]. RNA cleaved by a piRNA-loaded RISC would in turn be recognized as a pre-piRNA by the presence of an appropriate 5’ end and then loaded into a RISC, ultimately cleaving more RNA and perpetuating the proposed ping-pong amplification loop [21,22]. A primary role of the piRNA pathway is believed to be the suppression of transposable elements in the germline. No definitive role has been established for piRNAs in *Drosophila* somatic cells, though a piRNA pathway that utilizes only Piwi, and not Aubergine or Argonaute 3 (Ago3), has been identified in *Drosophila* somatic follicle cells [28,29]. Recent evidence, however, indicates significant differences between the piRNA pathways present in *Drosophila* and those of mosquitoes [30].

Mosquitoes have a larger repertoire of Piwi-clade Ago proteins (Ago3 and seven Piwis) than *Drosophila*, all of which appear to be expressed in the soma [30,31]. Multiple studies have also demonstrated a class of 24 to 30 nt viral small RNAs in mosquito tissues and cells possessing hallmarks of production by a ping-pong dependent piRNA pathway [30,32]. In contrast with the canonical piRNA pathway, in which piRNAs are expressed predominantly in the germline, viral piRNAs are produced in the soma [30]. Also in contrast with the canonical piRNA pathway, dsRNAs may be involved in the biogenesis of these viral piRNAs [30]. Several lines of evidence also suggest that this somatic piRNA pathway is an important antiviral defense in mosquitoes. First, the production of piRNAs from viral RNA, described in *Drosophila* OSS cells as well as various mosquito cell lines and tissues, is itself suggestive of an antiviral function [30,32,33]. Second, in contrast to the enhanced disease phenotype associated with Dcr2 null mutant flies infected with viral pathogens, an antiviral immune response directed by virus-derived piRNAs modulates the pathogenicity of alphavirus infection in Dcr2 null mutant mosquito cells [30]. Finally, knocking down components of the piRNA pathway in mosquito cells enhances alphavirus replication [34].

## 5. Mammalian RNAi

While the antiviral role of RNAi has been well established in plants and invertebrates, a role for RNAi in the antiviral defense of vertebrates remains the subject of debate. While RNAi machinery is conserved among plants, invertebrates, and mammals, it does not appear to have the same central role in the antiviral immunity of mammals that it does in other eukaryotes. Similar to plants and invertebrate animals, dsRNA induces an innate immune response; however, the mammalian response is mediated by interferon (IFN) rather than RNAi [35]. Nevertheless, transfection of synthetic siRNAs into mammalian cells induces an RNAi response capable of targeting mammalian viruses [36,37]. Several groups have used next generation sequencing to look for evidence of naturally occurring viral siRNAs in mammalian cells infected with a wide range of viruses, but the results have been ambiguous [38,39]. One study, which was particularly broad in scope, demonstrated accumulation of virus-derived small RNAs (vsRNAs) in various mammalian cell lines when infected with several different RNA viruses [40]. Although these vsRNAs were present in relatively low abundance, they did share characteristics of viral siRNAs identified in other organisms [40]. However, the putative viral siRNAs identified in these cells did not exhibit a predominant 21 nt or 22 nt length, nor could they be shown to associate with a specific Ago family member, although they did associate with multiple Ago proteins [40]. This study also did not specifically demonstrate the potential of the putative viral siRNAs to mediate a specific antiviral immune response in mammalian cells [40]. Determining the role of the mammalian RNAi machinery in antiviral immunity is complicated by the fact that dsRNA (>30 base pairs in length) induces IFN, leading to the transcription of a large number of IFN-stimulating genes (ISGs). Some ISGs are also capable of degrading viral mRNAs into smaller RNA products. For example, Girardi *et al.* recently described a stable class of alphavirus-derived small RNAs, 21 to 28 nt in length, generated by the endonuclease RNase L in human and monkey cell lines [41]. It also remains possible that there are other novel viral small RNA products that have yet to be described in mammalian cells.

Two high profile studies, released concurrently within the last year, support an antiviral role for RNAi in mammalian cells. In one of these studies, Li *et al.* examined small RNA production in mammalian cells and young mice challenged with Nodamura virus (NoV; *Nodaviridae*: *Alphanodavirus*), which encodes a strong VSR [42]. In both cells and mice, the VSR appeared to reduce accumulation of small RNAs derived from the virus. Ablating VSR expression or its activity resulted in accumulation of vsRNAs possessing several properties of canonical siRNAs [42]. In the other study, Maillard *et al.* examined small RNA production in undifferentiated mouse embryonic stem cells (mESCs) infected with either NoV or encephalomyocarditis virus (EMCV; *Picornaviridae*: *Cardiovirus*) [43]. Similarly, these authors also described the accumulation of vsRNAs with hallmarks of canonical siRNAs in the mESCs. The vsRNAs were not observed in Dicer knockout cells and increased in abundance with loss of the NoV-encoded VSR, coinciding with a replication-deficient phenotype. Importantly, replication of the mutant NoV was rescued in RNAi-deficient mouse cells. Both studies support a role for RNAi in the antiviral immunity of mammalian cells [43]. However, the results of these two studies appear to conflict with the results of another study using recombinant vesicular stomatitis viruses (VSV; *Rhabdoviridae*: *Vesiculovirus*) expressing either a VSR or an inhibitor of the IFN response [44]. The authors found that the virus expressing the VSR actually replicated to lower levels than the wild type virus, whereas the virus that interfered with the host IFN response replicated to higher levels than the wild type virus [44]. Replication of the virus expressing the VSR was rescued in host cells lacking a functional IFN pathway [44]. Analysis of small RNA populations in infected cells indicated that the presence of a VSR negatively impacted viral replication by reducing or eliminating transcriptional regulation of the host cell’s IFN response [44]. These results are in agreement with earlier findings by Seo *et al.*, which provide the basis for a model proposing that the RNAi machinery of mammalian cells serves as a negative regulator of the IFN response [45]. In this model, RISC-mediated RNA silencing inhibits the expression of ISGs associated with apoptosis and cell proliferation, suggesting that these IFN-mediated responses are exacerbated by expression of a VSR [45]. Consequently, these studies support a different role for mammalian RNAi than the direct antiviral function suggested by the other studies. Though seemingly at odds, these findings are not necessarily mutually exclusive as it is possible that the mammalian RNAi response is pathogen-specific or plays a direct antiviral role only under certain conditions. The work described in Maillard *et al.* supports a model in which mammalian RNAi initially plays a direct antiviral role in undifferentiated cells such as mESCs, but is eventually replaced by the IFN response as cells begin to differentiate, possibly assuming an indirect role as a negative regulator of ISGs [43].

While many questions remain regarding the role of mammalian small RNA pathways in antiviral defense, it is clear that the presence of conserved antiviral silencing pathways in invertebrate organisms plays an important role in the transmission of agents of human and animal disease. Arthropod-borne viruses (arboviruses) are responsible for a broad spectrum of human disease, including arthritis, hemorrhagic fever, and encephalitis. In nature, arboviruses rely upon blood feeding arthropod vectors such as mosquitoes for transmission between susceptible vertebrate hosts, meaning that arboviruses have evolved to replicate in both vertebrate and invertebrate cells. While infection of the vertebrate host might be associated with a wide range of symptoms or even death, the virus must be capable of establishing a persistent infection in the invertebrate vector host. The virus is ingested by the invertebrate host during blood feeding and initially replicates in the midgut before spreading to other tissues. Once present in the salivary glands, the virus can be transmitted to a new vertebrate host during subsequent blood feeding. If the infection is too virulent, however, the invertebrate host may not efficiently transmit the virus to a susceptible vertebrate host. Conversely, replication that is too attenuated may result in the virus being cleared by the invertebrate host’s immune response before transmission can occur.

## 6. Identifying Suppression of RNAi

In order to overcome the small RNA-directed immune response of the host, viruses have evolved methods of suppressing RNAi, resulting in what is often referred to as an evolutionary arms race between virus and host [46]. While VSRs appear to be ubiquitous in plant viruses [47], VSRs have only been identified in a relatively small number of insect viruses (Table 1) [48]. Some of the earliest VSRs identified were found in potato Y virus (PYV; *Potyviridae*: *Potyvirus*) and cucumber mosaic virus (CMV; *Bromoviridae*: *Cucumovirus*) using assays designed to detect a reversal of gene silencing [49,50]. In such an assay, a transgenic host expressing a post-transcriptionally silenced reporter gene is infected with a virus. If RNAi is suppressed, expression of the previously silenced reporter gene will result in an observable phenotype. A variation on this type of assay employs co-transfection of a reporter plasmid and a plasmid expressing a putative VSR. The suppressor function of some mammalian virus proteins has been inferred from studies expressing the putative VSR as an isolated protein with a second reporter construct [48]. While this type of assay is a useful tool, this approach may not always accurately reflect the authentic function of the protein during viral infection. Studies of individual viral proteins are unlikely to replicate the spatial and temporal levels of protein expression during an infection, nor do such studies consider interactions with other viral proteins that might alter function [48]. Finally, the role of mammalian RNAi machinery in antiviral defense remains unresolved, leaving open the possibility that some of the proteins found to possess suppressor activity in mammalian viruses actually evolved to counter IFN-mediated antiviral responses induced by dsRNA. For example, although the LaCrosse virus (LACV; *Bunyaviridae*: *Orthobunyavirus*) NSs protein has been shown to inhibit RNA silencing, NSs-deficient mutants of LACV exhibit growth deficiencies in IFN-competent mouse embryo fibroblast (MEF) cells, but replicate to wild type levels in MEF cells lacking the type I IFN receptor [51].

Another popular approach is to test for dsRNA binding activity, as some of the most well characterized VSRs have been found to interfere with the activity of Dcr2 or the loading of siRNAs into the RISC by binding dsRNA. One common method is the electrophoretic mobility shift assay (EMSA), also known as a gel shift assay, which uses gel electrophoresis to distinguish a slower migrating RNA/protein complex from unbound RNA. Unfortunately, this technique is not capable of establishing that the dsRNA binding capability is due to the proposed suppressor activity of the candidate VSR and not some other function of the protein. This is one of the challenges of working with viral proteins, which are so often multifunctional in nature. Genetic rescue experiments have also successfully identified VSR proteins in many plant and insect viruses [48]. Genetic rescue experiments are based on the idea that VSRs are dispensable in systems deficient in RNAi. For example, experiments in which replication of the insect flock house virus (FHV; *Nodaviridae*: *Alphanodavirus*) deficient in the expression of its VSR, the B2 protein, was rescued in Dcr2 and Ago2 null mutant flies provided definitive proof of the B2 protein’s suppressor activity [52,53,54]. Replication of a virus in which the putative VSR is absent or genetically ablated will be limited, or prevented altogether, in cells possessing a functional antiviral response; however, replication of the virus will be rescued in RNAi defective host cells. The challenge to this approach again stems from the frequently multifunctional nature of viral proteins, many of which are critical to the replication of the virus. Thus, removing or mutating the VSR may negatively impact other functions necessary for replication of the virus, making it impossible to use this approach. Hence the appeal, and often necessity, of conducting *in vitro* studies with isolated proteins. Genetic rescue can also be accomplished by utilizing a recombinant virus in which the VSR has been replaced with that of a heterologous candidate protein [55,56]. Such recombinant viruses may also be used in combination with RNAi null mutant cells or organisms [57].

## 7. Viral Suppressors of RNAi

**Figure 1:**
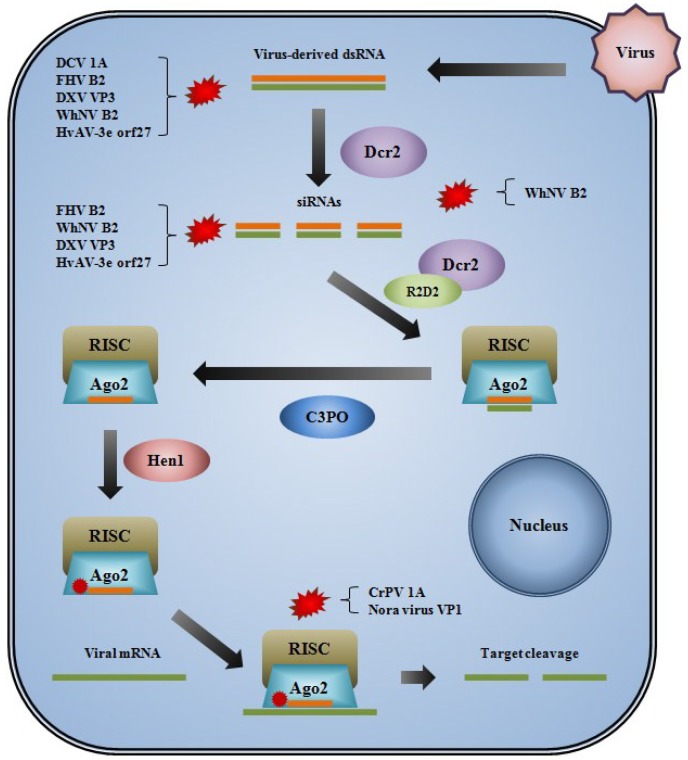
Diagram illustrating a general overview of the siRNA-mediated antiviral response in an insect cell and the points at which VSRs of insect-infecting viruses are known or thought to interfere with the pathway. Viral dsRNA triggers the siRNA pathway and is processed into siRNAs by Dcr2.The siRNAs are loaded into the RISC by Dcr2 and R2D2. The passenger strand is removed by C3PO and the guide strand is methylated by Hen1. The guide strand binds complementary viral dsRNA, which is then cleaved. FHV B2, WhNV B2, and DXV VP3 have been shown to bind small and long dsRNA, whereas DCV 1A binds only long dsRNA. HvAV-3e orf27 may competitively degrade dsRNA to prevent siRNA formation and/or loading. WhNV B2 potentially interacts with Dcr2 directly to inhibit function. CrPV 1A and Nora virus VP1 appear to directly interfere with the function of Ago2/RISC to prevent target cleavage.

Consistent with the limited coding capacity of viral genomes, VSR proteins are often encoded in overlapping reading frames or suppressor activity will have evolved in unrelated viral proteins with other functions [47]. As a result, viral suppressors differ greatly with respect to sequence, structure, and mode of action (Figure 1). Two of the most well characterized viral suppressors are RNA binding proteins that shield dsRNA produced during viral infection from Dicer processing and RISC assembly. The Cymbidium ringspot virus (CymRSV; *Tombusviridae*: *Tombusvirus*) P19 protein dimerizes to form a “molecular caliper” that measures the length of a dsRNA [58,59,60]. A P19 dimer exhibits the greatest binding affinity for dsRNAs that are 21 nt in length, with progressively weaker binding to shorter or longer dsRNAs [58,59]. Thus, P19 specifically interferes with the incorporation of siRNA duplexes into the RISC [58,59]. More recently, P19 was also shown to specifically induce expression of miR168 in CymRSV-infected plants, an activity that appears to be independent of the protein’s ability to bind dsRNA. Increased expression of miR168 was shown to negatively regulate Ago1 levels in the virus-infected plants, demonstrating that P19 has multiple suppressor functions [61,62]. The molecular mechanism by which the FHV B2 protein mediates suppression is more general. Indiscriminate binding of dsRNA by the B2 protein interferes with both the processing of long dsRNAs by Dcr2 and the incorporation of siRNA duplexes into the RISC [57,63,64]. Recently, the VP3 proteins of mosquito, *Drosophila*, and avian entomobirnaviruses (*Birnaviridae*) were shown to suppress RNAi through a similar mechanism [55,56], while the *Drosophila* C virus (DCV; *Dicistroviridae*: *Cripavirus*) 1A protein has been shown to inhibit the Dcr2-mediated production of siRNAs by binding long dsRNA, but not short dsRNA, including siRNAs [54]. A possible third mechanism of action for B2-mediated suppression of RNAi was recently proposed. A study examining Wuhan nodavirus (WhNV; *Nodaviridae*: *Betanodavirus*), a relative of FHV, suggests that WhNV B2 may also bind directly to Dcr2 [65]. Deletion mutants of WhNV B2 indicate that the protein’s C-terminal domain interacts directly with the RNase III and Piwi-Argonaut-Zwille domains of Dcr2, inhibiting the nuclease’s ability to process dsRNA and interfering with loading of siRNAs into the RISC [65].

Although dsRNA binding is essential to the suppressor activity of these proteins, it is not yet clear if this is a general feature of most VSR proteins. For example, the VSR 1A protein encoded by cricket paralysis virus (CrPV; *Dicistroviridae*: *Cripavirus*) does not bind dsRNA or siRNAs, but instead appears to exert an inhibitory effect on RNA silencing through a direct interaction with Ago2 [66]. Similarly, the P38 capsid and VSR protein encoded by turnip crinkle virus (TCV; *Tombusviridae*: *Carmovirus*) has been shown to directly interact with Ago1 of *Arabidopsis thaliana* [67]. In this case, P38 mimics cellular proteins containing glycine/tryptophan (GW/WG) residue repeats. Previously characterized host-encoded GW/WG repeat proteins have been found to promote the assembly and/or action of RISCs [68]. Viral mimicry of these proteins by P38 is thought to interfere with RISC assembly or function through a tight interaction with Ago proteins [67]. As the requirement for GW/WG-rich proteins in RISC assembly and function appears to be widespread in plants and animals, pathogen mimicry of these proteins might prove to be a broadly conserved strategy for modulating RNA-based host immune responses. This is supported by identification of GW/WG motifs in plant and vertebrate viruses, some of which correspond to previously identified virulence factors [67,69,70]. Other suppressors that interact directly with RNA silencing components include the Nora virus (*Picornaviridae*) VP1 protein [71], the cucumber mosaic virus (CMV; *Bromoviridae*: *Cucumovirus*) 2b protein [72], the polerovirus (*Luteoviridae*) P0 protein [73], and the cauliflower mosaic virus (CaMV; *Caulimoviridae*: *Caulimovirus*) P6 protein [74]. Another mechanism of suppression has been identified among the ascoviruses, a family of DNA viruses that infects lepidopteran insects [75]. Open reading frame 27 (orf27) of the *Heliothis virescens ascovirus* (HvAV-3e; *Ascoviridae*: *Ascovirus*) genome encodes a RNase III that is highly conserved among ascoviruses and appears to play an important role in viral replication [75]. Evidence suggests that HvAV-3e orf27 serves as a VSR by competitively degrading the same dsRNA substrate targeted by host Dcr2, or possibly degrading siRNAs, preventing the formation of a mature RISC. 

**Table 1:** Known or putative VSRs of insect viruses.

Suppressor	Virus	Mechanism(s) of Action	Evidence
1A	Drosophila C virus (DCV; *Dicistroviridae*: *Cripavirus*)	Binds long dsRNA [54]	Reporter silencing assays
			Dicing assays
			Loss of function mutants
VP3	Drosophila X virus (DXV; *Birnaviridae*: *Entomobirnavirus*)	Binds long and short dsRNA [56]	Reporter silencing assays
			RNase protection assays
			Gel shift assays
			Loss of function mutants
			Genetic rescue experiments
B2	Flock house virus (FHV; *Nodaviridae*: *Alphanodavirus*)	Binds long and short dsRNA [57]	Reporter silencing assays
			Gel shift assays
			Dicing assays
			Genetic rescue experiments
B2	Wuhan nodavirus (WhNV; *Nodaviridae*: *Betanodavirus*)	Binds long and short dsRNA [65] Binds to Dcr2 [65]	Co-immunoprecipitation assays
			RNase protection assays
			Loss of function mutants
1A	Cricket paralysis virus (CrPV; *Dicistroviridae*: *Cripavirus*)	Interferes with Ago2 function [66]	Reporter silencing assays
			Gel shift assays
			Dicing assays
			Slicing assays
			Co-immunoprecipitation assays
			Loss of function mutants
VP1	Nora virus (*Picornaviridae*?)	Interferes with Ago2 function [71]	Reporter silencing assays
			Gel shift assays
			Slicing assays
orf27	Heliothis virescens ascovirus (HvAV-3e; *Ascoviridae*: *Ascovirus*)	Competitive degradation of dsRNA [75]	Reporter silencing assays
			Knockdown assays
			Northern blots
			qPCR

## 8. Suppressors of Invertebrate RNAi Encoded by Arboviruses

**Figure 2:**
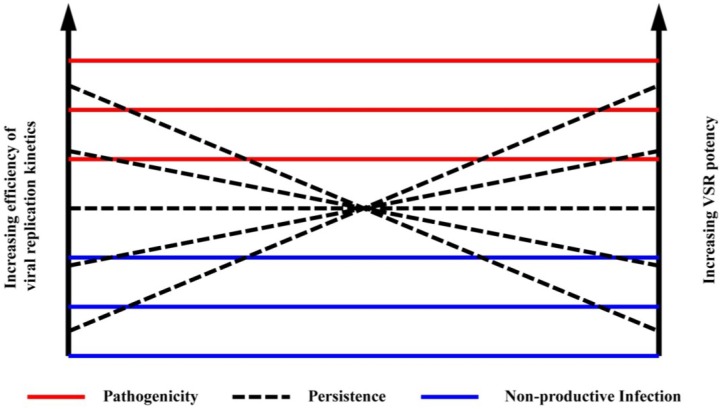
Diagram illustrating how the proposed relationship between VSR potency and the efficiency of viral replication kinetics affects the outcome of viral infection. As illustrated by the red lines, pairing a potent VSR with high replication kinetics would most likely result in a pathogenic infection, whereas pairing a weak VSR with poor replication kinetics is likely to result in a non-productive infection, as indicated by the blue lines. The establishment of a persistent infection would be facilitated by pairings indicated with dashed lines, such as the combination of a potent VSR with slow replication kinetics. A virus with slow replication kinetics might require a more potent VSR to facilitate persistent infection, whereas a virus with fast replication kinetics might require only a weak VSR, if any, in order to achieve persistence.

Evidence indicates that RNA silencing plays an important role in the transmission of mosquito-borne viruses. The majority of mosquito-borne viruses are classified in one of three families that differ greatly with respect to genomic organization and replication strategy: *Togaviridae*, * Flaviviridae*, * and Orthobunyaviridae*. These viruses are maintained in nature through alternating virus replication in susceptible vertebrate and mosquito hosts. While infection of the vertebrate host is typically associated with pathology and disease, a persistent non-lethal infection is established in the insect host. The mechanisms by which this occurs are not well understood, but we have previously shown that an antiviral response directed by siRNAs is essential to this process [76]. Expression of the FHV B2 protein from recombinant alphaviruses dramatically alters the pathogenic outcome of the virus infection in a mosquito host, resulting in rapid and complete mortality [76]. As the potency of the VSRs encoded by some insect viruses has been correlated with the virulence of natural infections [66], our previous results do not necessarily preclude the presence of VSR proteins in the genomes of mosquito-borne viruses. However, persistent infection likely requires that the replication kinetics of the virus be balanced against VSR potency and the antiviral response of the host. Replication kinetics describes the speed and efficiency with which the virus is able to carry out various processes within the host cell. How efficient a virus is at co-opting the replicative machinery of the host cell or the efficiency with which the viral proteins are expressed is likely to have selected for VSR potency. In this model (Figure 2), viruses with slower replication kinetics may require a more potent VSR to achieve the necessary equilibrium required for persistent infection, whereas viruses with more rapid replication kinetics may derive a selective advantage by encoding a less potent VSR, minimizing any negative impact the virus may have on vector fitness. This model assumes an equally robust antiviral response in each case. For example, evidence suggests the piRNA pathway plays a more important antiviral role in mosquitoes than it does in fruit flies [30], and this needs to be considered when interpreting the results of studies examining candidate arboviral VSR proteins in the genetic model organism. While it can be useful to study VSR proteins in isolation, in the context of heterologous virus genomes, or in non-native hosts, it is important to keep in mind the factors contributing to the ongoing co-evolution of the virus and its natural host.

One of the earliest arboviral suppressor candidates (Table 2) was identified in Bunyamwera virus (BUNV; *Bunyaviridae*: *Orthobunyavirus*), which is the prototype bunyavirus. Experiments comparing the replication of wild type virus and a recombinant deletion mutant of the non-structural protein NSs of BUNV have demonstrated a role in pathogenesis in the mammalian host, despite being dispensable to viral replication [77]. NSs inhibits protein synthesis and interferes with activation of the IFN-mediated immune response in mice, but does not inhibit protein synthesis in mosquitoes [78]. A study by Szemiel *et al.* found that deletion of NSs was less detrimental to virus replication in mosquito cells lacking a functional siRNA pathway than it was to replication in cells possessing a fully functional siRNA pathway [79]. However, the mutant virus also replicated differently in the two different Dcr2 null cell lines used. The presence of an antiviral piRNA response in these cell lines may have contributed to the differences. Although it was necessary to use multiple mosquito cell lines in these studies, consequently making interpretation of the results less clear than rescue in isogenic models, the mutant virus was also found to be less successful at infecting adult mosquitoes than the wild type virus [79]. It has also been suggested that the NSs of the closely related LACV may act as a VSR [80]. A biological mechanism for the putative VSR proteins remains unclear, but if their role in suppressing the antiviral immune response of the vector is confirmed, it is interesting that these proteins have evolved similar functions in both vertebrate and invertebrate hosts.

Another VSR candidate has been identified in the genomes of DENV and West Nile virus (WNV; *Flaviviridae*: *Flavivirus*). Flaviviruses produce a subgenomic RNA from the 3’ untranslated portion of the genome that has been shown to be highly resistant to nuclease-mediated degradation [81]. Interestingly, a role for this subgenomic flavivirus RNA (sfRNA) in the pathogenicity of these viruses in mammalian hosts has also been demonstrated [81]. Schnettler *et al.* used reporter gene silencing assays to demonstrate suppression of RNAi by sfRNA in arachnid, insect, and mammalian cell lines [82,83]. Based on an observed inhibition of *in vitro* processing of long dsRNAs by Dicer in the presence of sfRNA, the authors postulated that the non-coding RNA might act as a competitive substrate for the RNase III enzyme. A recent study demonstrates a role for the DENV type 2 sfRNA as a molecular sponge or decoy for the mammalian RNA binding proteins G3BP1, G3BP2, and CAPRIN1, which interferes with the translation of ISGs and the establishment of an antiviral state [84]. These findings appear to be consistent with this model, although specific binding of sfRNA to Dicer has not yet been interrogated. Molecular decoys derived from nucleic acid sequences have also been proposed as a possible mechanism by which alphaviruses might evade the antiviral response in the invertebrate host [85].

Kakumani *et al.* have proposed a third arboviral suppressor candidate [86]. The DENV NS4B protein also was initially identified as an inhibitor of the mammalian IFN-mediated immune response [87]. Kakumani *et al.* demonstrated by *in vitro* dicing assays that NS4B is capable of interfering with the processing of siRNAs, apparently by a mechanism other than dsRNA binding. Reporter gene silencing assays in mammalian cells challenged with NS4B deletion mutants suggest that transmembrane domains 3 and 5 are involved in the reported VSR activity. These domains also appear to be different from those involved in inhibition of the mammalian immune response; however, the specific mechanism by which NS4B interferes with Dicer activity remains unclear. Evidence of VSR activity by NS4B in the vector host is also lacking. 

**Table 2:** Putative VSRs of arboviruses.

Suppressor	Virus	Mechanism(s) of Action	Evidence
NSs	Bunyamwera virus (BUNV; *Bunyaviridae*: *Orthobunyavirus*)	Unknown [79,80]	Reporter silencing assays
	LaCrosse virus (LACV; *Bunyaviridae: Orthobunyavirus*)		Loss of function mutants
sfRNA	Dengue virus (DENV; *Flaviviridae*: *Flavivirus*)	Competitive substrate for Dicer [82,83]	Reporter silencing assays
	West Nile virus (WNV; *Flaviviridae*: *Flavivirus*)		Gel shift assays
	Langat virus (LGTV; *Flaviviridae: Flavivirus*)		Dicing assays
	Tick-borne encephalitis virus (TBEV; *Flaviviridae: Flavivirus*)		
NS4B	Dengue virus (DENV; *Flaviviridae*: *Flavivirus*)	Unknown [86]	Reporter silencing assays
			Gel shift assays
			Dicing assays

It remains unclear how mosquito-borne viruses modulate the vector’s RNA-based immune response to establish productive infection, and interrogating these viruses for the presence of suppressor proteins presents a number of challenges. As mentioned above, studies involving isolated proteins may either exaggerate or fail to detect suppressor activity, while a lack of mutants deficient in key components of the RNA silencing pathway prohibits genetic rescue experiments in the natural invertebrate host. The presence of multiple small RNA-based antiviral immune pathways in the vector host may also present challenges, and it will be interesting to see if mosquito-borne viruses have evolved VSRs that suppress an antiviral response mediated by piRNAs. Studies that have identified VSR proteins in plant and insect viruses have often been guided by prior experimental evidence that identified a particular viral protein as a virulence factor before any suppressor activity was suspected. Studies identifying genetic determinants of virulence in mosquito-borne viruses have largely been conducted in vertebrate hosts. While it seems counterintuitive that these studies would be relevant to the invertebrate host, where infection with the virus tends to be avirulent, each of the candidate VSRs identified in arboviral genomes thus far have previously been associated with virulence in mammalian hosts. It will be interesting to see if this trend continues, and raises a number of interesting questions about how and why these functions evolved in the same viral proteins. It will also be interesting to see how much overlap exists in the mechanisms by which these proteins mediate interference with the RNAi-based immunity of invertebrates and the IFN-based immunity of mammals; perhaps even more so as questions regarding the role of RNAi in the innate immune responses of mammalian cells are resolved.
